# Patterns of antibiotic resistance genes and virulence factor genes in the gut microbiome of patients with osteoarthritis and rheumatoid arthritis

**DOI:** 10.3389/fmicb.2024.1427313

**Published:** 2024-11-20

**Authors:** Yaqi Guo, Hang Feng, Lin Du, Zhenghong Yu

**Affiliations:** Department of Surgery of Spine and Spinal Cord, Henan Provincial People's Hospital, People's Hospital of Zhengzhou University, People's Hospital of Henan University, Zhengzhou, China

**Keywords:** gut microbiome, antibiotic resistance genes, virulence factor genes, rheumatoid arthritis, osteoarthritis, non-invasive diagnostic

## Abstract

**Background:**

The gut microbiome compositions of osteoarthritis (OA) and rheumatoid arthritis (RA) patients have been revealed; however, the functional genomics, particularly antibiotic resistance genes (ARGs) and virulence factor genes (VFGs), have not yet been explored.

**Methods:**

We used gut metagenomic data to elucidate the distribution of ARGs and VFGs. Building on these differences in gut microbiome, we developed a diagnostic model using a random forest classifier based on ARG and VFG abundances.

**Results:**

Our results indicated that both OA and RA patients exhibit significantly higher alpha diversity in ARGs, as measured by observed genes, the Shannon index, and the Simpson index, compared to healthy controls. However, this increased diversity is not significantly different between OA and RA patients. In contrast, VFGs showed higher diversity in RA patients than in healthy individuals, which was not as pronounced in OA patients. An analysis of the top 20 ARGs and VFGs revealed a largely similar composition between the three groups, with notable exceptions of certain genes that were uniquely enriched in either OA or RA patients. This suggests unique microbial patterns associated with each condition. Our beta diversity analysis further demonstrated distinct distributions of ARG and VFG profiles across the three groups, with several genes significantly enriched in both OA and RA patients, indicating potential markers for these diseases. The model achieved high accuracy (74.7–83.6%) when distinguishing both OA and RA from healthy controls using ARG profiles and substantial accuracy using VFG profiles.

**Conclusion:**

These results support the potential of ARGs and VFGs as reliable biomarkers for diagnosing OA and RA.

## Introduction

Osteoarthritis (OA) and rheumatoid arthritis (RA) are two of the most common joint diseases (Grillet et al., 2023). OA is a degenerative joint disease, while RA is an autoimmune disease. Although OA and RA are incurable (Yap et al., 2018; Yu et al., 2021), studies have indicated that both osteoarthritis and rheumatoid arthritis are closely linked to gut microbiota (Yu et al., 2021; Zhao et al., 2022). Trillions of symbiotic microbes (bacteria, fungi, and archaea) reside in the human gastrointestinal tract, maintaining a close relationship with the human body and contributing to the preservation of our health. Yu et al. (2021) found that several microbes were causally associated with diverse joint OA. In addition, Wells et al. (2020) revealed that the presence of *Prevotella* spp. was positively associated with RA. Furthermore, a recent study revealed that *Escherichia coli* and *Streptococcus bovis* could promote RA progression by enhancing ascorbate degradation (Zhao et al., 2022). Interestingly, sustained increases in intestinal permeability induce intestinal microbial invasion of the joint synovial fluid (Cheng et al., 2022), highlighting the importance of the gut microbiome and its functional genome in the development of OA and RA.

Using amplicon sequencing technology (Chiang et al., 2019; Huang et al., 2020), the composition of gut microbes can be analyzed, and shotgun metagenomic data can explore the functional genome of gut microbes (Qin et al., 2010), especially certain key genes, such as antibiotic resistance genes (ARGs) (Alcock et al., 2020) and virulence factor genes (VFGs) (Liu et al., 2022). Antibiotics are often used after joint replacement surgery (Yates and American Association of Hip and Knee Surgeons Evidence-Based Medicine Committee, 2018). It is important to understand how these antibiotics interact with gut microbes. VFGs have been used to study the molecular pathogenesis of RA.

However, early and non-invasive diagnosis of the disease is crucial. Detecting OA in its early stages is challenging due to the limited correlation between pain and structural degradation (Yu et al., 2021). Similarly, accurate biomarkers for RA play a critical role in the early detection of the disease and monitoring its activity and progression (Yap et al., 2018). A previous study has examined the use of gut microbial markers to diagnose immune diseases, including RA, with an accuracy of 81 and 86% (Forbes et al., 2018). The diagnosis of osteoarthritis is more frequent using image-based learning techniques (Alshamrani et al., 2023; Jena et al., 2021) and mass spectrometry-based biomarkers of the joint synovial fluid (Haartmans et al., 2021), and methods based on gut microbes need to be further explored. Understanding the abundance and diversity of ARGs and VFGs in the gut microbiome of OA and RA may be of great significance. Whether the functional genome of the intestinal microbiome can be used for disease diagnosis needs to be further explored.

In this study, our primary goal was to reveal the composition of ARGs and VFGs in the gut microbiome of OA and RA patients and explore the use of these features as biomarkers for the diagnosis of OA and RA. Our study offers a non-invasive and promising way to diagnose patients early and understand disease progression.

## Materials and methods

### Data collection and quality control

A total of 76 gut microbiome samples from RA patients, 19 samples from OA patients, and 26 samples from healthy controls were collected based on the National Genomics Data Center (project accession number CRA004348) (Zhao et al., 2022; Cheng et al., 2022). The shotgun metagenomic sequencing data is available for download. The inclusion and exclusion criteria for cohorts have been described in previous studies, with brief, healthy individuals in good health condition with no gastrointestinal diseases and RA/OA individuals with no other co-morbidity (Zhao et al., 2022). Trimmomatic v0.39 (Bolger et al., 2014) was used to trim adapter sequences (ILLUMINACLIP:TruSeq3-PE-2.fa) and filter out low-quality bases using the parameters (2:30:10:8:true, TRAILING:20, MINLEN:60). Post-quality control, the sequencing reads were processed further to eliminate contamination from human genomic DNA using Bowtie v2.4.4 (Langmead and Salzberg, 2012). This decontamination step was achieved by aligning reads against the human reference genome T2T-mY-rCRS (Nurk et al., 2022), and then the sequencing data were used to annotate functional genomes. The data analysis workflow is illustrated in Supplementary Figure S1. First, we collected and conducted quality control on the metagenomic data, then analyzed the composition of ARGs and VFGs, identified potential biomarkers, and constructed a predictive model for the disease using random forests.

### ARG and VFG annotation and abundance evaluation

After excluding sequences of human genomic origin, we performed sequence alignment with the Comprehensive Antibiotic Resistance Database (CARD) (Alcock et al., 2020) (available at https://card.mcmaster.ca/download) and the Virulence Factor Database (VFDB) (Liu et al., 2022) (accessible via http://www.mgc.ac.cn/VFs/main.htm), using Bowtie v2.4.4 (Langmead and Salzberg, 2012) and Samtools v1.13 (Li et al., 2009). To accurately determine the prevalence of ARGs and VFGs, adjustments were made for both the depth of sequencing and the lengths of the individual genes (Chang et al., 2021; Ma et al., 2020). This process resulted in normalized relative abundance data for the ARGs and VFGs, which is essential for the downstream analytic procedures.

### Alpha and beta diversity calculation

In R v4.3.2, alpha diversity metrics, including the observed genes, Shannon index, and Simpson index, were derived using the vegan package (Zapala and Schork, 2006). Bray–Curtis distances were calculated directly from the ARG and VFG abundance profiles. Principal coordinates analysis (PCoA) was performed using the ade4 package.^1^ Furthermore, the vegan package was used to perform the Adonis test (permutations = 999).

### Diagnostic model construction

A random forest classifier (Rigatti, 2017) was used to develop a predictive model for RA and OA. We focused on genomic features from the gut microbiome, including ARGs and VFGs. The research process begins with loading the dataset, which encompasses a variety of genomic features alongside corresponding disease outcomes. This dataset is bifurcated into predictor variables (X), containing the genomic features, and response variables (y), denoting the disease outcomes. To isolate features strongly associated with the disease outcome, Pearson’s correlation coefficients are computed for each feature against the target variable, and the top 2000 features with the strongest correlations are retained for subsequent analysis. The dataset is then partitioned into a training set, which holds 80% of the data, and a testing set, which contains the remaining 20%. A diagnostic model is constructed using the random forest classifier, with key parameters such as the count of decision trees (n_estimators) and the maximum tree depth (max_depth) fine-tuned to enhance test set performance. Model efficacy is evaluated using a receiver operating characteristic (ROC) curve and area under the curve (AUC) scores, which provide insight into the model’s discriminatory capabilities.

### Statistical analysis

The statistical analysis was performed in R version 4.3.2. To assess the distinctions in the abundance of ARGs and VFGs, the Wilcoxon rank-sum test was applied, and the Benjamini–Hochberg (BH) method was implemented for *p*-value adjustment, considering *p*-adjust <0.05 as statistically significant. The construction of boxplots, barplots, and PCoA plots was facilitated using the ggplot2 package. In addition, the generation of ROC curves was accomplished with the aid of the pROC package.

## Results

### Alpha diversity based on ARG and VFG profiles was increased in the gut microbiome of OA and RA patients compared to healthy controls

**Figure 1:**
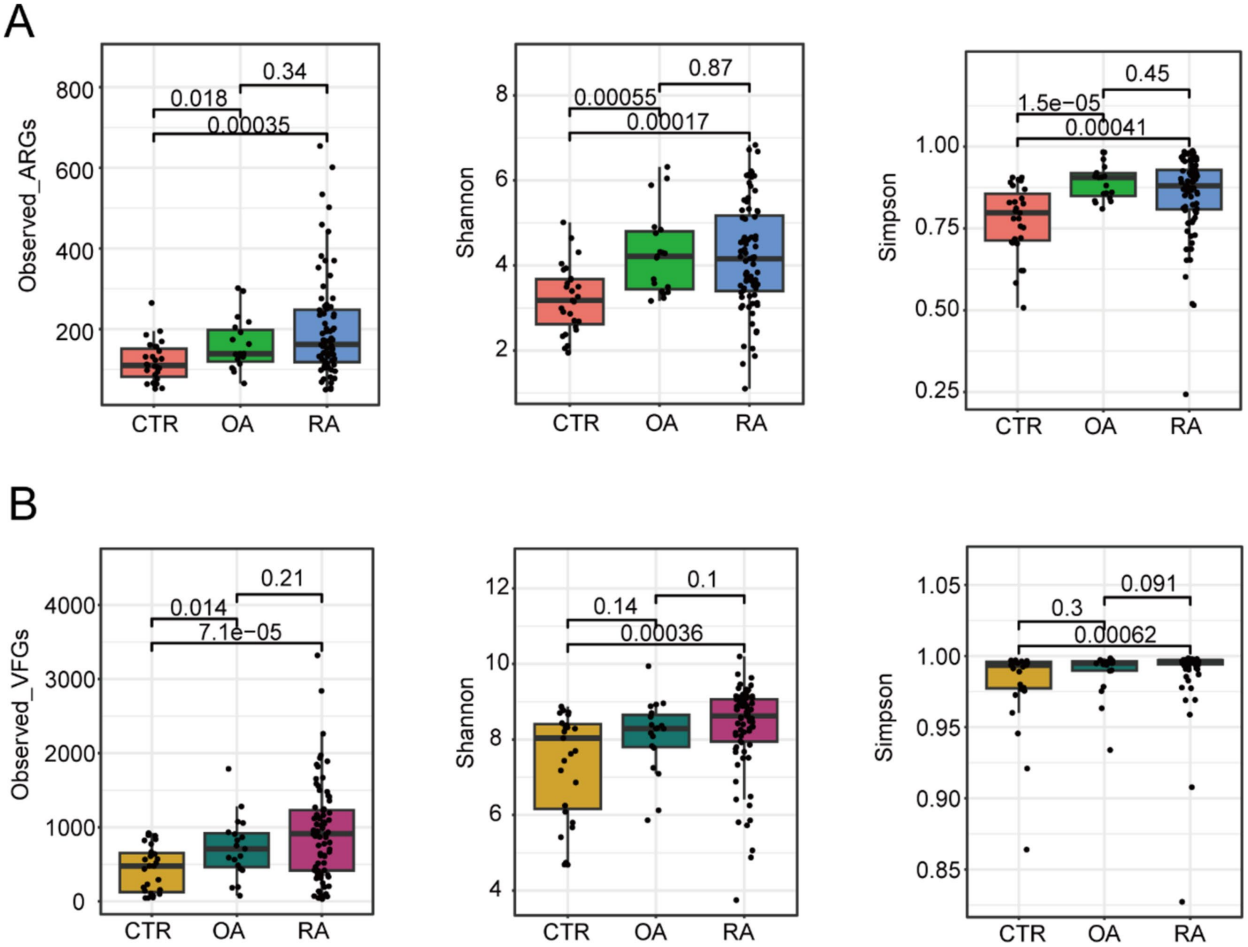
Different alpha diversity among CTR, OA, and RA groups. **(A)** Alpha diversity based on ARG profiles. **(B)** Alpha diversity based on VFGs profiles. Wilcoxon test was used to calculate the *p*-value, and a *p*-value of <0.05 was considered significant. CTR, healthy control; OA, osteoarthritis; RA, rheumatoid arthritis.

To elucidate the differences between ARGs and VFGs driven by the gut microbiomes of patients with OA and RA, we first investigated the alpha diversity based on the abundances of ARGs and VFGs, which included metrics such as observed genes, Shannon index, and Simpson index (Figures 1A,B). We discovered that the diversity of ARG compositions in OA and RA patients was significantly higher than that in healthy individuals (observed genes: *p* = 0.018, 0.00035, Shannon index: 0.00055, 0.00017, Simpson index: 1.5e-5, 0.00041, Figure 1A), with no significant difference observed between OA and RA patients (observed genes: *p* = 0.34, Shannon index: *p* = 0.87, Simpson index: *p* = 0.45, Figure 1A). Subsequently, we found that the diversity of VFGs in RA patients was also higher than in healthy individuals (observed genes: *p* = 7.1e-5, Shannon index: *p* = 0.00036, Simpson index: *p* = 0.00062, Figure 1B), whereas such distinction was only observed in metric observed genes between OA and healthy individuals, with the other indices showing no difference (observed genes: *p* = 0.014, Shannon index: *p* = 0.14, Simpson index: *p* = 0.3, Figure 1B). No difference was detected between OA and RA (observed genes: *p* = 0.21, Shannon index: *p* = 0.1, Simpson index: *p* = 0.091, Figure 1B). These findings suggested that gut microbiota in OA and RA patients may have a certain pattern of ARG and VFG compositions.

### Different dominant ARGs and VFGs in the gut microbiome of OA and RA patients and healthy controls

**Figure 2:**
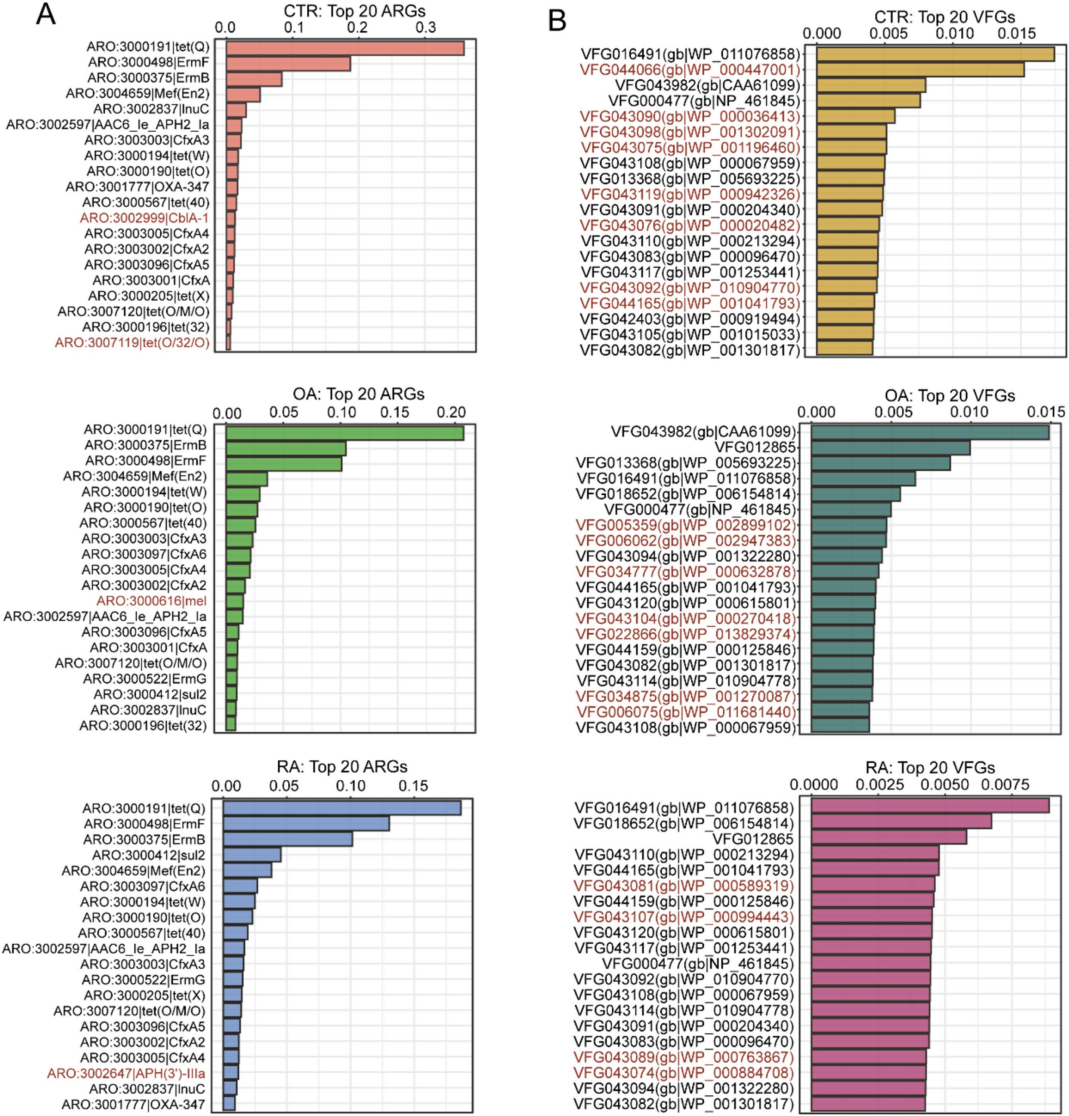
Top 20 ARGs and VFGs based on relative abundance among CTR, OA, and RA groups. **(A)** Top 20 ARGs among CTR, OA, and RA groups. **(B)** Top 20 VFGs among CTR, OA, and RA groups. Genes marked with red were unique in specific groups. CTR, healthy control; OA, osteoarthritis; RA, rheumatoid arthritis.

To further understand the composition of ARGs and VFGs driven by the gut microbiomes of patients with OA, RA, and healthy controls, we focused on the top 20 features ranked in each group. These features could reflect the differences in dominant genes between each group. Initially, we found that the composition of the top 20 ARGs between OA, RA, and healthy controls was quite similar (Figure 2B), with only 1–2 gene differences, mainly concentrated in antibiotic-related genes such as tet(Q), ErmF, and ErmB. In Figure 2B, we highlighted the genes that were only ranked in the top 20 within specific groups. For instance, CblA-1 and tet(O/32/O) were higher in the healthy group than in the OA and RA groups. Meanwhile, mel was only ranked in the top 20 within the OA group, and APH(3′)-lla was observed to be in the top 20 exclusively in the RA group. Subsequently, the ranking of VFGs distinctly differed from the pattern of ARGs, with most ARGs showing a greater distribution across groups and each group possessing 4–8 unique major VFGs (Figure 2B). The above results suggest that there may be significant differences in the distribution of ARGs and VFGs across the three groups.

### Different distribution of ARG and VFG profiles in the gut microbiome of OA and RA patients and healthy controls

**Figure 3:**
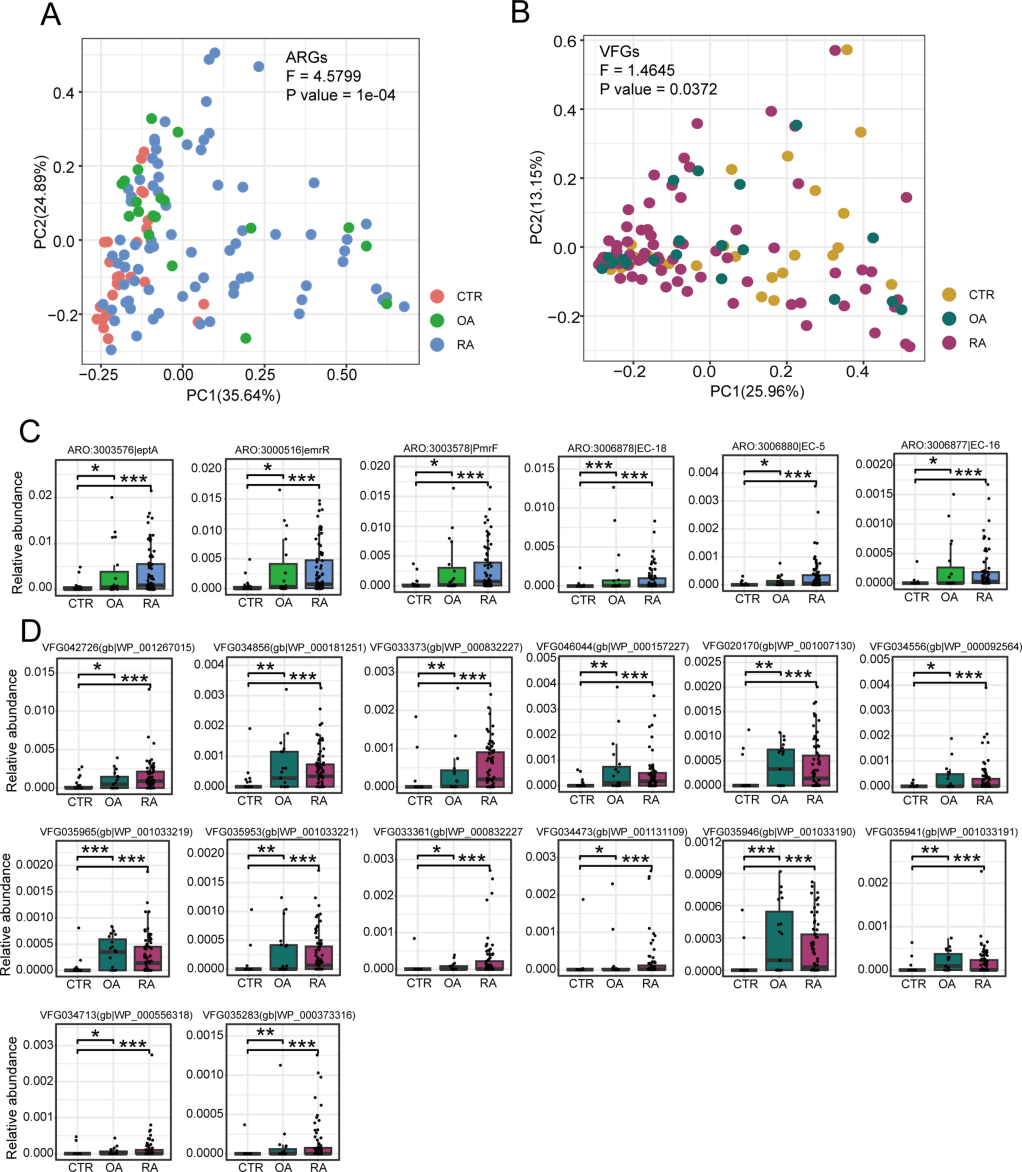
Comparison of beta diversity among the CTR, OA, and RA groups, along with the differential genes identified between OA and CTR, as well as RA and CTR. **(A)** Beta diversity among three groups based on ARG profiles. **(B)** Beta diversity among three groups based on VFG profiles. **(C)** Enriched ARGs in OA and RA groups compared to healthy controls. **(D)** Enriched VFGs in OA and RA groups compared to healthy controls. CTR, healthy control; OA, osteoarthritis; RA, rheumatoid arthritis.

We further compared the differences in the distributions of ARGs and VFGs among the three groups using beta diversity. We calculated the Bray–Curtis distances between samples based on the abundance data of ARGs and VFGs. We observed that the distributions of ARGs and VFGs were significantly different among the three groups (ARGs: *p* = 1e-4, *F* = 4.5799; VFGs: *p* = 0.0372, *F* = 1.4645, Figures 3A,B). This suggests that there might be specific differences in the abundance of certain ARGs and VFGs, further strengthening the notion that certain ARGs and VFGs are significantly enriched in both OA and RA. A total of 6 ARG genes were found to be enriched in both OA and RA, namely eptA, emrR, PmrF, EC-18, EC-5, and EC-16 (Figure 3C). Meanwhile, 14 VFGs were found to be significantly enriched in both OA and RA (Figure 3D). Although the significant differential genes are not the primary top 20 genes in Figure 2, they are still important biomarkers that may help us distinguish between OA and RA.

### OA and RA diagnosis model based on ARGs and VFGs

**Figure 4:**
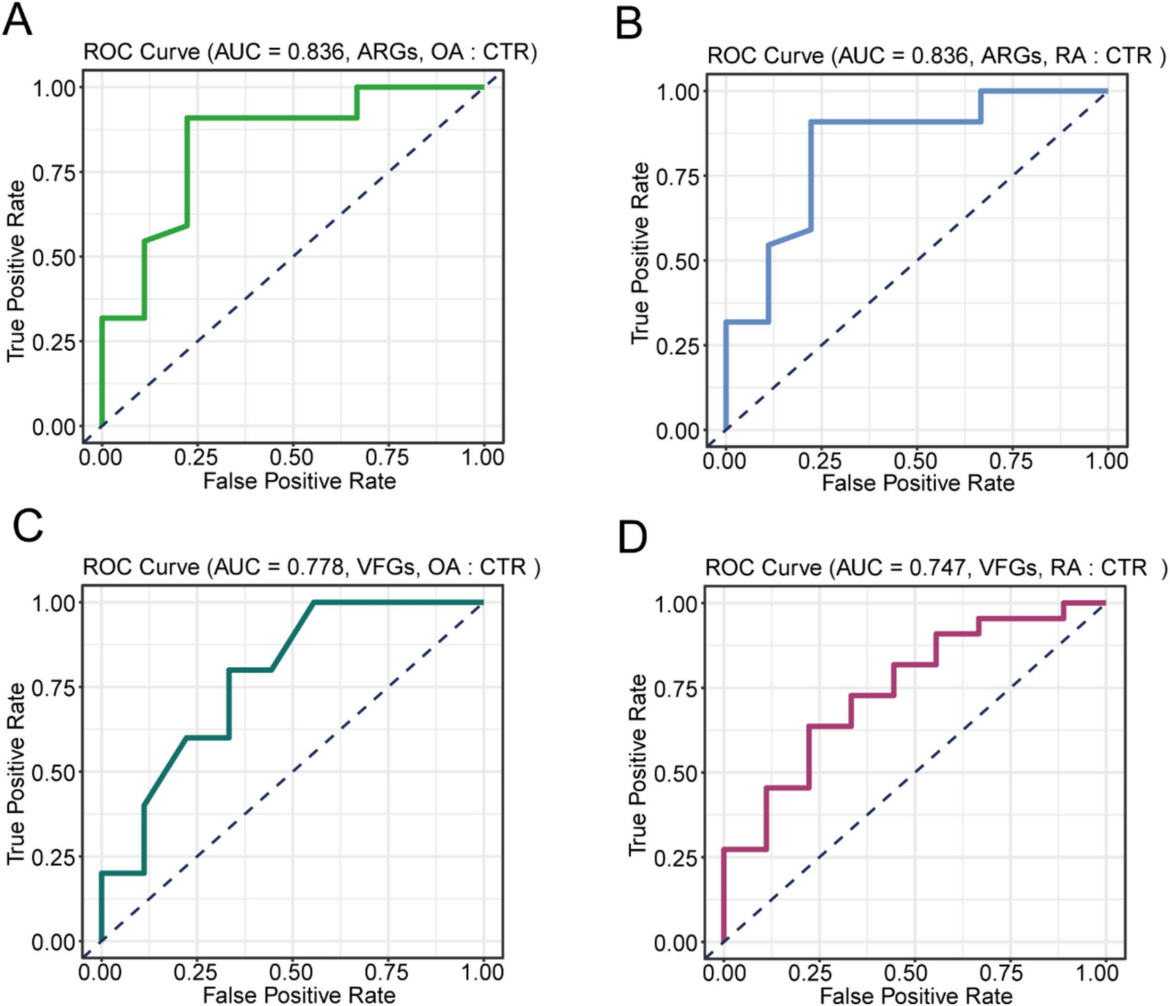
Construction of the diagnostic model. **(A)** Performance of model based on ARGs distinguish on OA and CTR. **(B)** Performance of model based on ARGs distinguish on RA and CTR. **(C)** Performance of model based on VFGs distinguish on OA and CTR. **(D)** Performance of model based on VFGs distinguish on RA and CTR. CTR, healthy control; OA, osteoarthritis; RA, rheumatoid arthritis.

Due to the significant differences in ARGs and VFGs found in the gut microbiomes of OA and RA patients, we further investigated the development of diagnostic models for OA and RA using these features. For this purpose, we used a random forest model based on the abundance of ARGs and VFGs. Initially, distinguishing OA patients from healthy controls using ARG profiles achieved an accuracy of 83.6% (Figure 4A). Similarly, the accuracy for distinguishing RA patients from healthy individuals was also 83.6% (Figure 4B). When using VFG profiles, the accuracy dropped to 77.8% for differentiating OA patients from healthy individuals (Figure 4C), and 74.7% for differentiating RA patients from healthy individuals (Figure 4D). These findings indicated that both ARGs and VFGs could serve as biomarkers for the diagnosis of OA and RA.

## Discussion

The human gut microbiota plays a vital role in modulating the immune response and inflammatory pathways, making it a promising target for studying OA and RA (du Teil Espina et al., 2019; Luo et al., 2023; Thompson et al., 2023). In this study, our findings revealed that individuals with OA and RA display a significantly elevated alpha diversity in ARGs or VFGs. Then, our beta diversity analysis further illustrated varying distributions of ARG and VFG profiles. The importance of ARGs and VFGs in gut microbiota cannot be overstated, particularly in the context of inflammatory conditions such as OA and RA. The presence of ARGs in the gut microbiome is a growing public health concern (Theophilus and Taft, 2023). As antibiotics are widely used in clinical practice, the selection pressure they exert on microbial communities can lead to the proliferation of resistant strains. This is particularly concerning in patients with OA, who often receive antibiotic treatment, such as tetracycline, which is commonly prescribed for managing symptoms (Platt et al., 2021; Daniels et al., 2022). In addition, previous findings indicated that therapeutic agents aimed at inhibiting eptA activity may enhance the effectiveness of polymyxins and aid the immune system in clearing infections (Samantha and Vrielink, 2020). The accumulation of ARGs not only hampers the effectiveness of antibiotic therapies but also poses a risk of systemic infections and further complicates disease management (Khan et al., 2024). In parallel, VFGs play a critical role in the pathogenicity of microorganisms, contributing to their ability to establish infections and evade host immune responses. Certain VFGs have been implicated in the development of chronic inflammatory diseases, including RA (Jin, 2024). By exploring these interactions, we can gain valuable insights into how microbial communities influence disease development and progression.

OA and RA are two common joint diseases that result in significant morbidity and disability. The traditional diagnostic approaches for OA and RA rely heavily on clinical symptoms (Alsayyad et al., 2021), imaging studies (Alshamrani et al., 2023; Jena et al., 2021), and serological markers (Haartmans et al., 2021; Gilbert et al., 2023), which may not always provide definitive and early diagnosis (Yap et al., 2018; Yu et al., 2021). To address the issue, we developed a model to detect OA and RA, and the accuracy of our model is approximately 74.7–83.6%. Although the current accuracy is lower than that of imaging studies in OA patients and microbial biomarkers in RA patients, studying the functional genes of intestinal microbes provides us with a deeper understanding of host–microorganism interaction mechanisms. Our findings underline the promising potential of ARGs and VFGs as reliable biomarkers for the diagnosis of OA and RA. Although our model successfully diagnoses the disease, the diagnostic model needs further validation and refinement to consider populations with different dietary habits and characteristics across different countries (Thomas et al., 2019; Wirbel et al., 2019). The integration of microbiome analysis into standard diagnostic protocols offers substantial clinical utility. Identifying specific ARG and VFG profiles in patients could lead to earlier and more accurate diagnoses, guiding personalized treatment plans that directly address the specific microbial imbalances driving disease. For instance, identifying patients with a particular set of microbial signatures could inform targeted antibiotic therapies or the use of microbiome-modulating treatments, such as probiotics or fecal microbiota transplants. However, our study cannot explain the cause of ARG and VFG enrichment and its impact on the host, which requires further investigation. Specifically, further investigations could focus on understanding how specific ARGs and VFGs influence immune responses, inflammatory pathways, and tissue remodeling in the context of joint diseases. In addition, research into the potential therapeutic implications of modulating gut microbiota through dietary interventions or probiotics may provide novel strategies for managing OA and RA.

## Conclusion

Our study demonstrates that patients with OA and RA exhibit significantly higher alpha diversity in ARGs than healthy controls, as evidenced by observed genes, the Shannon index, and the Simpson index. However, this increased diversity is not significantly different between OA and RA patients. In terms of VFGs, RA patients show greater diversity than healthy individuals, a trend that is less pronounced in OA patients. Analysis of the top 20 ARGs and VFGs revealed largely similar compositions among the three groups, with specific genes uniquely enriched in either OA or RA patients, suggesting distinct microbial patterns associated with each condition. In addition, beta diversity analysis illustrated notable differences in ARG and VFG profiles among the groups, highlighting several genes that are significantly enriched in both OA and RA patients, which may serve as potential markers for these diseases. Importantly, our predictive model demonstrated high accuracy in distinguishing both OA and RA patients from healthy controls based on ARG profiles, as well as substantial accuracy using VFG profiles.

## Data Availability

Publicly available datasets were analyzed in this study. This data can be found at: All shotgun metagenomic sequence can be download Genome Sequence Archive (GSA) section of the National Genomics Data Center (project accession number CRA004348).

## Footnotes

^1^ https://github.com/adeverse/ade4
