# Supplementary figures and images for: Patterns of antibiotic resistance genes and virulence factor genes in the gut microbiome of patients with osteoarthritis and rheumatoid arthritis
Source: Front Microbiol. 2024 Nov 20;15:1427313. doi: 10.3389/fmicb.2024.1427313 (PMC11615078; doi:10.3389/fmicb.2024.1427313)

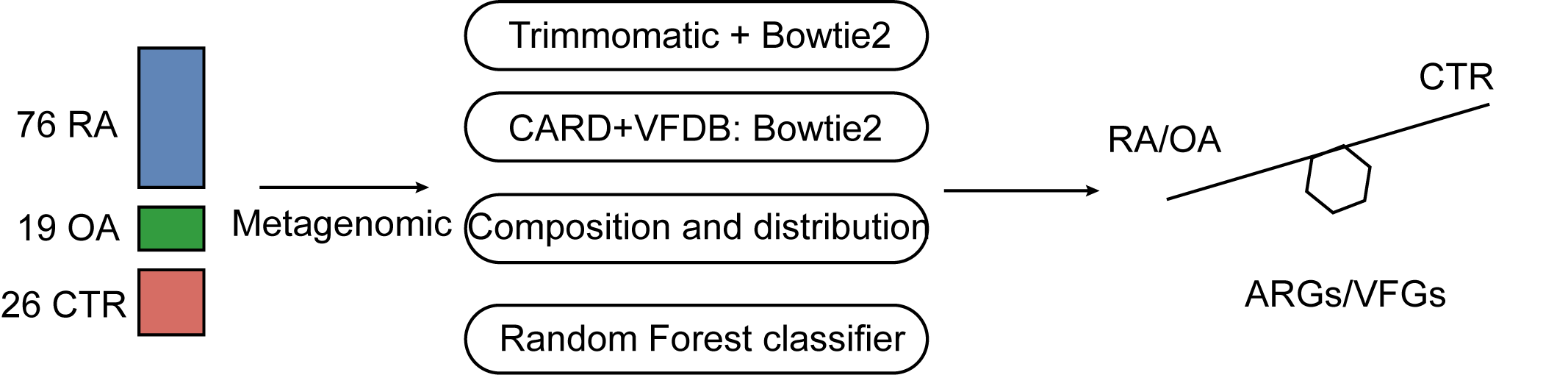

Supplement: Supplementary file 1 [file Image_1.tif]
